# The L-shape relationship between hemoglobin, albumin, lymphocyte, platelet score and the risk of diabetic retinopathy in the US population

**DOI:** 10.3389/fendo.2024.1356929

**Published:** 2024-05-10

**Authors:** Ranran Ding, Yusong Zeng, Zhimei Wei, Zitong He, Zhixin Jiang, Jinguo Yu, Caiyun You

**Affiliations:** ^1^ Department of Ophthalmology, Tianjin Medical University General Hospital, Heping District, Tianjin, China; ^2^ Tianjin Medical University, Heping District, Tianjin, China; ^3^ Tianjin Eye Hospital, Nankai University Affiliated Eye Hospital, Clinical College of Ophthalmology, Tianjin Medical University, Tianjin Eye Institute, Tianjin Key Laboratory of Ophthalmology and Visual Science, Tianjin, China

**Keywords:** HALP score, diabetic retinopathy, biomarker, NHANES, diabetes complication

## Abstract

**Background:**

The primary aim of this study was to investigate the correlation between diabetic retinopathy (DR) and the HALP score (hemoglobin, albumin, lymphocyte, and platelet) in individuals with diabetes within the United States population.

**Methods:**

This cross-sectional investigation was based on the National Health and Nutrition Examination Survey (NHANES) database from 2003-2018. The following module calculated the HALP score: HALP score = [lymphocytes (/L) × hemoglobin (g/L) × albumin (g/L)]/platelets (/L). By performing the receiver operating characteristic (ROC) analysis, the optimal cutoff value of HALP was ascertained. Restricted cubic splines (RCS), multivariable logistic regression analysis, sensitivity analysis, and subgroup analysis were conducted to evaluate the effect of the HALP score on DR patients. Finally, the decision curve analysis (DCA) and clinical impact curve (CIC) were conducted to estimate the predictive power and clinical utility of the HALP score with clinical indicators.

**Results:**

According to the cutoff value (42.9) determined by the ROC curve, the participants were stratified into a lower HALP group (HALP_low_) and a higher HALP group (HALP_high_). An L-shaped relationship between HALP score and DR risk was presented in the RCS model (P for nonlinearity <0.001). The DR risk sharply decreased with the increase of HALP, and the decline reached a plateau when HALP was more than 42.9. After fully adjustment, the multivariate logistic regression analysis found that HALP_low_ was an independent risk factor for DR (OR = 1.363, 95% CI: 1.111-1.671, P < 0.001). Besides, sensitivity analysis showed consistent results. Furthermore, the combination of HALP score and clinical indicators demonstrated predictive power and clinical utility, as shown by the ROC curve, DCA, and CIC.

**Conclusion:**

The HALP score has an L-shaped correlation with the risk of DR, and thus, the HALP score may contribute to the timely intervention of diabetes patients.

## Highlights

- Limited studies have focused on the relationship between diabetic retinopathy (DR) and the hemoglobin, albumin, lymphocyte, platelet (HALP) score. We were the first team to demonstrate a negative association between lower HALP score and the prevalence of DR.
- An L-shaped correlation between HALP and DR occurrence was also initially observed in our findings. Moreover, diabetic patients with HALP score <49.2 were found to have a significantly increased risk of DR.
- We examined a large sample size, which represented 19.3 million residents in the United States.

## Introduction

Diabetes mellitus (DM) is an escalating worldwide public health concern projected to impact around 700 million individuals by 2045 (1). Diabetic retinopathy (DR), a visually impairing condition associated with DM, remains the primary cause of avoidable vision impairment in the working-age population. It is estimated that approximately 160.5 million people with DM will suffer from DR in 2045 (1). Therefore, exploring new predictors of DR occurrence may help in the early identification and intervention of DR, which will play a crucial role in mitigating the visual impairment or loss associated with DM (2).

The etiology of DR is complex and multifaceted, with diverse factors involved, such as dyslipidemia and chronic inflammation (3). Previous evidence demonstrated that combinations of the hematological indices, such as neutrophil-to-lymphocyte ratio (NLR) and platelet-to-lymphocyte ratio (PLR), were regarded as being associated with DR incidence (4, 5). However, there are disagreements in some findings in terms of the correlation between NLR, PLR, and DR (6–9). The inconsistent conclusions make people realize that the use of single parameters representing inflammation status does not meet the requirements of clinical practice; therefore, multi-parameter combinations are necessary to be explored.

Increasing evidence supports that nutrition status also plays a role in the initiation and progression of DR in addition to inflammation (10–12). The combination of hemoglobin, albumin, lymphocyte, and platelet (HALP) score, as a novel immune-nutritional marker, provides insights into the chronic inflammation and immunological condition of the patients (13), which is relatively more stable than single blood parameters. In a cross-sectional study, researchers found that low hemoglobin concentrations are associated with a higher risk of DR (14). Serum albumin, representing nutritional state and metabolic demands (15), was found to have a quantitatively significant negative correlation with DR (16). Recently, much clinical research suggested that a low HALP score was indicative of a poor prognosis in multiple tumors (17–20). The latest studies have suggested that the HALP score is also related to dyslipidemia (21), which is a commonly acknowledged risk factor for DR. Nevertheless, the available evidence concerning the association between the HALP score and the occurrence of DR is extremely restricted.

To fill the research gap, this study investigated the relationships between DR and the HALP scores in a nationally representative sample of individuals with diabetes in the United States.

## Materials and methods

### Data source

**Figure 1:**
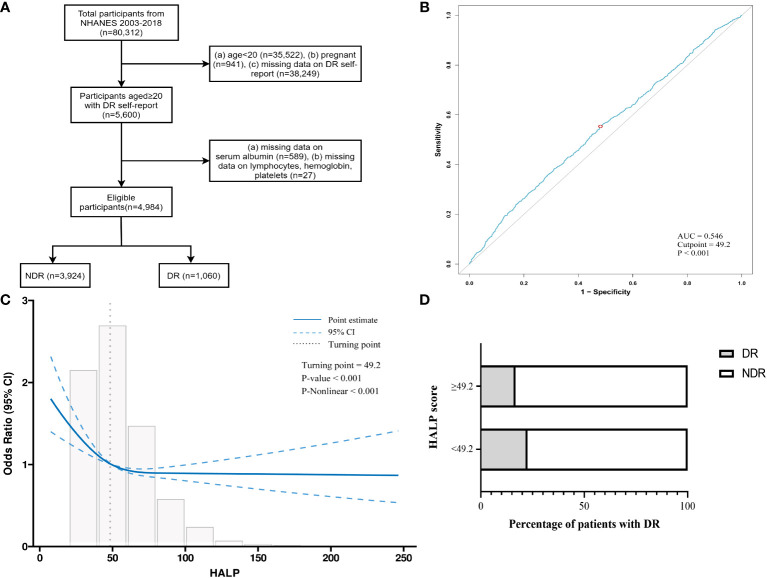
Study design and cutoff determination. **(A)** Flow diagram for research. **(B)** The cutoff of HALP score for the prediction of DR elevated by receiver operating characteristic curve (AUC=0.546, cutpoint=49.2, p<0.001). **(C)** The “L-shape” relationship between HALP score and DR based on restricted cubic splines (turning-point=49.2, p-nonlinear<0.001). **(D)** Stacked bar graphs of DR proportions in different HALP groups. AUC, Area under curve.

The National Health and Nutrition Examination Survey (NHANES), created by the National Center for Health Statistics (NCHS), is a series of publicly accessible cross-sectional surveys aiming to be representative of the US general population (https://www.cdc.gov/nchs/nhanes/). NCHS granted the study procedures of the Ethics Review Board (Protocol #98-12, #2005-06, #2011-17, #2018-01). Informed consent of the participants was obtained before collecting any data. All interviews and examinations were conducted under the guidance of the NHANES protocol. In this study, eight cycles of the NAHNES database were used (2003-2018), the selection process of which was depicted in **Figure 1A**. The exclusion criteria were: (a) age < 20 years (n = 35,522); (b) pregnant (n = 941); (c) without DR self-report (n = 38,249); (d) missing data on serum albumin (n = 589); and (e) missing lymphocyte, hemoglobin or platelet data (n = 27). Finally, 4,984 individuals participated in the investigation.

### Measurement of serum albumin and blood lymphocyte, hemoglobin, platelet count

Lymphocytes, hemoglobin, and platelets were derived from the complete blood count (CBC) using the Beckman-Coulter method of sizing and counting. Serum albumin was measured based on the bromcresol purple (BCP) dye approach in the NHANES database. The following module calculated the HALP score: HALP score = [lymphocytes (/L) × hemoglobin (g/L) × albumin (g/L)]/platelets (/L) (22).

### Ascertainment of DM and DR

DM was briefly defined by the Standards of Medical Care in Diabetes (23): (a) FPG ≥ 126mg/dL (7.0mmol/L), (b) 2-h OGTT ≥ 200mg/dL (11.1mmol/L), (c) HbA1c ≥ 6.5% (47.5mmol/L), (d) antidiabetic or insulin therapy, (e) who replied “yes” to the question “Did the doctor tell you that you have diabetes?” DR patients were those who replied “yes” to the question, “Has a doctor ever told you that diabetes has affected your eyes or that you had retinopathy?” The diabetes duration was calculated by: the claimed age when interviewing minus the age at first diagnosis of diabetes, and then separated into two categories: ≤ 10 years and > 10 years. Glycemic was assigned as excellent (HbA1c < 7%) or bad (HbA1c ≥ 7%) glucose management.

### Assessment of covariates

The selected demographic variables included age, gender, race, and education level. Examination and laboratory covariates for this study included body mass index (BMI), glycosylated hemoglobin A1c (HbA1c), low-density lipoprotein cholesterol (LDL-C), and high-density lipoprotein cholesterol (HDL-C). Self-reported daily habits and health state were also taken into account, including alcohol consumption, smoking status, and medicine history.

Dyslipidemia is frequently distinguished by three lipid abnormalities (24), namely: (a) increased levels of triglycerides (≥ 150 mg/dL), (b) increased levels of tiny LDL-C particles (≥ 130 mg/dL), and (c) decreased levels of HDL-C (< 40 mg/dL for men; <50 mg/dL for women). Besides, people who were prescribed medication for dyslipidemia were also considered. Hypertension was determined as a blood pressure measurement over 140/90 mmHg measured on three consecutive occasions, a related medicine history, or a professional diagnosis.

Smoking history was categorized based on self-report in the following manner: (a) non-smokers: individuals who have never consumed 100 cigarettes during their lives; (b) former smokers: individuals who previously smoked over 100 cigarettes but have quit smoking; (c) current smokers: individuals who have a history of current smoking. Alcohol consumption was assessed by a 24-hour food recall.

### Statistical analyses

The analysis of statistics was performed using Stata 16.0, R software (version 4.2.2), and MSTATA software. To ensure that the estimates could be representative of the general U.S. population, weighted samples, as well as the stratification and clustering of the design, were taken into consideration in all analyses conducted in accordance with centers for disease control (CDC) guidelines. To compare the disparities in baseline characteristics between the NDR and DR groups, continuous variables were expressed as means ± standard error (SE), and categorical variables were presented as proportions. When comparing the differences in continuous variables between patients with NDR and DR, a weighted t-test was applied for continuous variables, while a weighted chi-square test was used for categorical variables. The cutoff value of HALP to predict DR in diabetes subjects was initially determined by the receiver operating characteristic (ROC) curve, and then the HALP score in all the individuals was evaluated by the restricted cubic spline (RCS) curve. Furthermore, three logistic regression analysis models were performed to assess the relationship between HALP and DR prevalence, and sensitivity analysis and subgroup analysis were further carried out. Finally, the decision curve analysis (DCA) and clinical impact curve (CIC) were conducted to estimate the predictive power and clinical utility of the HALP score. A two-sided p < 0.05 was considered significant.

## Results

### Characteristics of the participants at baseline

Totally, 4,984 NHANES diabetes patients were enrolled in this study, representing 19.3 million individuals in the USA. The baseline characteristics of all the eligible individuals were displayed in **Table 1**, including 3,924 patients without DR and 1,060 patients with DR, and the weighted prevalence of DR was 19.57%. Totally, the average age was 59.6 ± 0.26 years, and 51.51% were males. In particular, in contrast to the NDR group, DR patients were more likely to have lower education level, longer diabetic duration, high levels of HDL-C, and HbA1c. The mean HALP score was 55.07 ± 0.78, and the DR patients tended to have lower HALP compared with NDR ones (50.67 ± 1.11 vs. 55.07 ± 0.78, P < 0.001).

**Table 1:** Baseline of participants with or without DR.

Characteristics	TOTAL (N=4984)	DR (-) (N=3924)	DR (+) (N=1060)	P-value
Age, years	59.94 ± 0.25	59.55 ± 0.28	59.88 ± 0.56	0.885
Gender, %				0.551
Male	51.42	51.63	50.57	
Female	48.58	48.37	49.43	
Race/Ethnicity, %				0.307
Mexican American	9.26	9.29	9.13	
Other Hispanic	5.37	5.29	5.71	
Non-Hispanic White	62.27	62.91	59.65	
Non-Hispanic Black	14.50	14.19	15.79	
Other (multi-racial)	8.59	8.32	9.73	
Education level				<0.001
Less than 9th grade	10.89	10.34	13.61	
9th-12th grade	13.41	12.93	15.41	
High School/GED	24.81	24.27	27.06	
Some college/AA	31.20	31.79	28.80	
College or above	19.52	20.60	15.09	
Missing	0.07	0.08	0.03	
Alcohol consumption, g/day	5.19 ± 0.40	5.38 ± 0.43	4.41 ± 0.99	0.186
Smoking status, %				0.512
non-smokers	48.85	48.53	50.18	
current-smokers	35.29	35.35	35.04	
former-smokers	15.86	16.12	14.79	
Diabetic duration, years	11.52 ± 0.23	10.28 ± 0.23	16.64 ± 0.62	<0.001
BMI, kg/m2	32.89 ± 0.15	32.88 ± 0.17	32.92 ± 0.34	0.896
BMI category				0.641
Normal (<25)	11.88	11.67	12.72	
Overweight (25-30)	26.04	26.03	26.09	
Obese (≥ 30)	62.08	62.30	61.19	
Hypertension, %				0.054
Yes	54.00	53.33	56.84	
No	46.00	46.68	43.16	
Hyperlipidemia, %				0.789
Yes	82.10	82.03	82.40	
No	17.90	17.97	17.60	
HbA1c, %	7.31 ± 0.03	7.21 ± 0.03	7.71 ± 0.07	<0.001
HDL-C, mg/dL	47.59 ± 0.27	47.34 ± 0.30	48.63 ± 0.59	0.010
HALP	55.07 ± 0.78	56.14 ± 0.93	50.67 ± 1.11	<0.001

Data was presented as means ± standard error (SE) or proportions.

BMI, Body mass index; HbA1c, Glycosylated hemoglobin A1c; HDL, High density lipoprotein; HALP, hemoglobin, albumin, lymphocyte, and platelet.

While no statistically significant disparity was observed in age, gender, race, body mass index, alcohol consumption, smoking history, hypertension presence, or hyperlipidemia presence among diabetes patients with or without DR.

### The relationships of HALP score with diabetic retinopathy

According to the analysis of the ROC curve, the optimal cutoff value for HALP to predict the DR prevalence in diabetes subjects was 49.2 (AUC = 0.546) (**Figure 1B**). Additionally, in the restricted cubic spline (RCS) model, a noteworthy nonlinear correlation was observed between HALP and DR risk (P-nonlinear < 0.001). An L-shaped association between HALP score and DR incidence was displayed in **Figure 1C**, and the inflection point of HALP for DR was also 49.2. Then, the relationships between HALP and DR were further analyzed by segmented logistic regression. The DR risk sharply decreased with the increase of HALP, and the decline reached a plateau when HALP was more than 42.9 (**Supplementary Table 1**).

Therefore, patients were categorized into two groups depending on their HALP scores: the higher HALP group (HALP_high_ ≥ 49.2) and the lower HALP group (HALP_low_ < 49.2). The comparison between DR patients and NDR patients was illustrated in **Figure 1D**, where the proportion of DR patients in the HALP_high_ group was smaller than that in the HALP_low_ group (16.74% vs. 22.53%, P < 0.001).

### Logistic regression analysis and sensitivity analysis

The results of logistic regression analyses evaluating the associations between HALP_low_ and DR in the diabetes population demonstrated that low HALP was associated with DR, regardless of other known factors (P < 0.05) (**Table 2**). Specifically, in the crude model (Model 1), a low HALP score was related to an elevated risk of DR (OR = 1.446, 95% CI: 1.188-1.760, P < 0.001). After several factors were adjusted (age, gender, race, BMI, education level, diabetic duration, and HbA1c level), a low HALP score was also presented as being related to the elevated risk of DR (OR = 1.364, 95% CI: 1.112-1.672, P =0.003). Considering extrema’s potential effects, a sensitivity analysis was performed to check the robustness of our results. After excluding samples with extreme HALP scores, similarly, sensitivity analysis indicated that the HALP_low_ group has a higher risk of DR prevalence in Model 3 (OR= 1.357, 95%CI: 1.107–1.664) (**Table 3**).

**Table 2:** Logistic regression analysis for the association between HALP and DR in various models.

	Model 1 OR (95%CI) P	Model 2 OR (95%CI) P	Model 3 OR (95%CI) P
HALP_high_	reference	reference	reference
HALP_low_	1.446(1.188–1.760) <0.001	1.453(1.185–1.781) <0.001	1.364(1.112–1.672)0.003
P trend	<0.001	<0.001	<0.001

Model 1: crude model, without any adjustments; Model 2: adjusted for age, gender, race; Model 3: based on model 2, further adjusted for diabetic duration, HbA1c, education level, BMI.

OR, Odds ratios; CI, Confidence intervals.

**Table 3:** Sensitivity analysis for the association between HALP and DR in various models.

	Model 1 OR (95%CI) P	Model 2 OR (95%CI) P	Model 3 OR (95%CI) P
HALP_high_	reference	reference	reference
HALP_low_	1.438(1.181–1.751)<0.001	1.445(1.178–1.772)<0.001	1.357(1.107–1.664)0.003

Model 1: crude model, without any adjustments; Model 2: adjusted for age, gender, race; Model 3: based on model 2, further adjusted for diabetic duration, HbA1c, education level, BMI.

OR, Odds ratios; CI, Confidence intervals.

### Stratification analysis of HALP score with diabetic retinopathy

**Figure 2:**
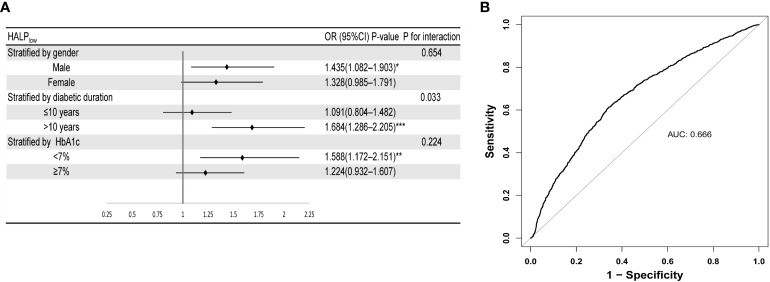
Robustness and diagnostic value of HALP. **(A)** Forest plot of the relationship of DR with HALP in different subgroups (after adjusting age, gender, race, BMI, education level, diabetic duration, and HbA1c level). **(B)** ROC curve of HALP score combined with clinical indicators (HbA1c level and diabetic duration) for predicting DR. Stratified by diabetes duration, HbA1c and gender. Subgroups were all adjusted for age, gender, race, diabetic duration, HbA1c, education level.

As shown in **Figure 2A**, further analysis was stratified by gender, diabetic duration, and HbA1c level. The findings from the subgroup analysis demonstrated persistent and favorable associations between HALP_low_ and DR risk across gender and HbA1c subgroups (P for interaction > 0.05). Notedly, HALP_low_ showed significantly higher prevalence of DR in the subgroups of longer diabetic duration (>10 years) (P for interaction = 0.033), with OR (95% CI) 1.684 (1.286-2.205).

### Predictive power of HALP score with clinical indicators for DR

To examine the diagnostic value of the HALP score combined with common clinical indicators for DR, we conducted ROC analysis. As depicted in **Figure 2B**, a combination of HALP score, HbA1c, and diabetic duration indeed results in a model with increased predictive performance (AUC=0.666). DCA investigated the potential clinical utility of the HALP score in predicting the risk of DR. DR demonstrated a favorable net clinical benefit within a threshold probability range of 7% to 40%, with the highest net benefit observed (**Supplementary Figure S1**). In addition, CIC was created as a visual tool to evaluate the concordance between prediction and observation of DR occurrences. As presented in **Supplementary Figure S2**, there were consistently more anticipated high-risk patients than real DR patients at the optimal threshold probability, with a satisfactory cost-benefit ratio.

## Discussion

To date, this was the first investigation into the potential association between HALP score and DR prevalence in diabetes patients, using the NHANES database based on a nationwide representative population scattered across the United States. The results suggested that a lower HALP score was a significant risk factor for DR, independent of other confounders. The DR risk for diabetes patients in the HALP_low_ group was 36% higher than that in the HALP_high_ group after adjusting for several factors. A similar conclusion was also drawn from sensitivity analysis, which represented that the findings were stable. Significantly, this research was the first to establish an L-shaped correlation between the HALP score and DR risk after adjusting for confounding variables. Additionally, a significant statistical trend was observed only when HALP was below 49.2. Specifically, HALP score showed a hazardous effect on DR occurrence when below 42.9 and then appeared relatively flat. Stratification analysis suggested that HALP_low_ was a reliable predictor of DR risk, thereby potentially aiding in the detection and monitoring of diabetes patients who are susceptible to DR, especially among patients with over 10 years of diabetic duration. Furthermore, the combination of HALP score and clinical indicators demonstrated predictive power and clinical utility, as shown by the ROC curve, DCA, and CIC.

The pathophysiology of DR is complex and multifactorial, involving chronic inflammation and oxidative stress. Accumulating studies have reported that chronic inflammation contributes to the upregulation of pro-inflammatory cytokines and chemokines, such as interleukin-1β (IL-1β) and vascular endothelial growth factor (VEGF), which can exacerbate the pathophysiological progression observed in DR, such as endothelial failure, leukocyte adhesion and infiltration, platelet activation, and neovascularization (25).

As a recognized indicator of inflammation, lymphocytes are actively involved in the elimination and repair of inflammation. Zhu et al. found that a lower lymphocyte percentage might potentially serve as a valuable diagnostic indicator for identifying the onset and progression of diabetic macular edema (DME) (26). Platelets play a crucial role in diabetes patients through heightened adhesion, activation, and aggregation of platelets due to disruptions in multiple signaling pathways and metabolic abnormalities such as hyperglycemia and dyslipidemia (27). Oxidative stress, systemic inflammation, reduced nitric oxide bioavailability, poor calcium metabolism, and increased phosphorylation and glycosylation of cellular proteins contribute to exacerbated platelet activation (28). This, in turn, leads to the occurrence of diabetic complications.

Serum albumin is rich in thiol groups that can effectively neutralize the majority of reactive oxygen in the blood (29), potentially aiding in protecting DR patients from oxidative damage. Furthermore, albumin has the ability to attach to advanced glycation end products (AGEs) formed through non-enzymatic glycation processes in a hyperglycemic environment (30). AGEs are strongly linked to the development of DR (31), and albumin can potentially reduce the harmful impact on the retina by binding to them (32). Hemoglobin, as an oxygen carrier, was correlated with more severe DR when it was in low concentration (33). Compared to red blood cells in the general population, those in diabetic individuals have less deformability and more capillary aggregation, making them more brittle and susceptible to breaking, leading to lower hemoglobin levels and potentially causing anemia (34). Hypoxia caused by anemia triggers the production of inflammatory mediators and vasoproliferative factors like VEGF and erythropoietin (35, 36). These substances can increase retinal vascular permeability and worsen DR (33). Moreover, anemia may lead to ischemia, which is believed to aggravate the progression of retinal hypoxia and further exacerbate the progression of DR (37).

The HALP score, a novel indication, is derived from the integration of the aforementioned four hematological parameters. This finding may be significant because it is cost-effective and easily assesses current inflammation and nutritional status, which can aid physicians in primary hospitals and outpatient clinics in evaluating DR occurrence especially when HALP score is lower than 42.9, and developing suitable treatment strategies through regulating hemoglobin, albumin, lymphocyte, and platelet.

This research exhibited some strengths and weaknesses. As far as our knowledge extends, this was the first investigation that assessed the potential correlation between HALP score and DR in patients with diabetes. Moreover, our study was a large sample size, which represented 19.3 million residents in the United States. Considering its cross-sectional and observational nature, some limitations existed in this study. First, this was a cross-sectional analysis, and causality cannot be extrapolated. Second, the history of DR in NHANES was just self-reported data and failed to involve the classification of DR. Third, the hemoglobin count, serum albumin levels, lymphocyte count, and platelet count were assessed during the examination. However, it failed to demonstrate how the HALP score exhibits dynamic changes over time. Consequently, prospective cohort investigations remain necessary to confirm the conclusions.

## Conclusion

The HALP score had an L-shaped association with the risk of diabetic retinopathy, suggesting possible predictive potential. Moreover, diabetes patients with a lower HALP score were more likely to have an increased risk of diabetic retinopathy, particularly among those with over 10 years of diabetic duration. Prospective studies are needed to prove its reliability and clinical utility.

## Data availability statement

The original contributions presented in the study are included in the article/**Supplementary Material**. Further inquiries can be directed to the corresponding author.

## Ethics statement

The NHANES protocols and testing procedures were all approved by the Institutional Review Board of the Centers for Disease Control and Prevention (Protocol #2005-06, Continuation of Protocol #2005-06, https://www.cdc.gov/nchs/nhanes/irba98.htm). All individuals who participated in the survey were provided with informed consent prior to their inclusion. The database included in this study underwent a process of de-identification, ensuring the removal of individually identifiable information and making it appropriate for non-human participant research. Therefore, no further ethical approval or consent to participate is needed.

## Author contributions

RD: Data curation, Formal Analysis, Methodology, Software, Writing – original draft. YZ: Investigation, Writing – original draft. ZW: Data curation, Writing – original draft. ZH: Conceptualization, Writing – review & editing. ZJ: Funding acquisition, Writing – review & editing. JY: Supervision, Writing – review & editing. CY: Funding acquisition, Writing – review & editing.

## Supplementary material

Supplementary Figure 1 Decision curve analysis of HALP score prediction model to estimate DR.

Supplementary Figure 2 Clinical impact curve of HALP score.

Supplementary Table 1 Effect of Standardized HALP Level on DR: Odds Ratios from Segmented Logistic Regression Analysis adjusted. *OR: Odds Ratio, CI: Confidence Interval, SD: Standard deviation.*
