# Supplementary figures and images for: The L-shape relationship between hemoglobin, albumin, lymphocyte, platelet score and the risk of diabetic retinopathy in the US population
Source: Front Endocrinol (Lausanne). 2024 May 10;15:1356929. doi: 10.3389/fendo.2024.1356929 (PMC11116578; doi:10.3389/fendo.2024.1356929)

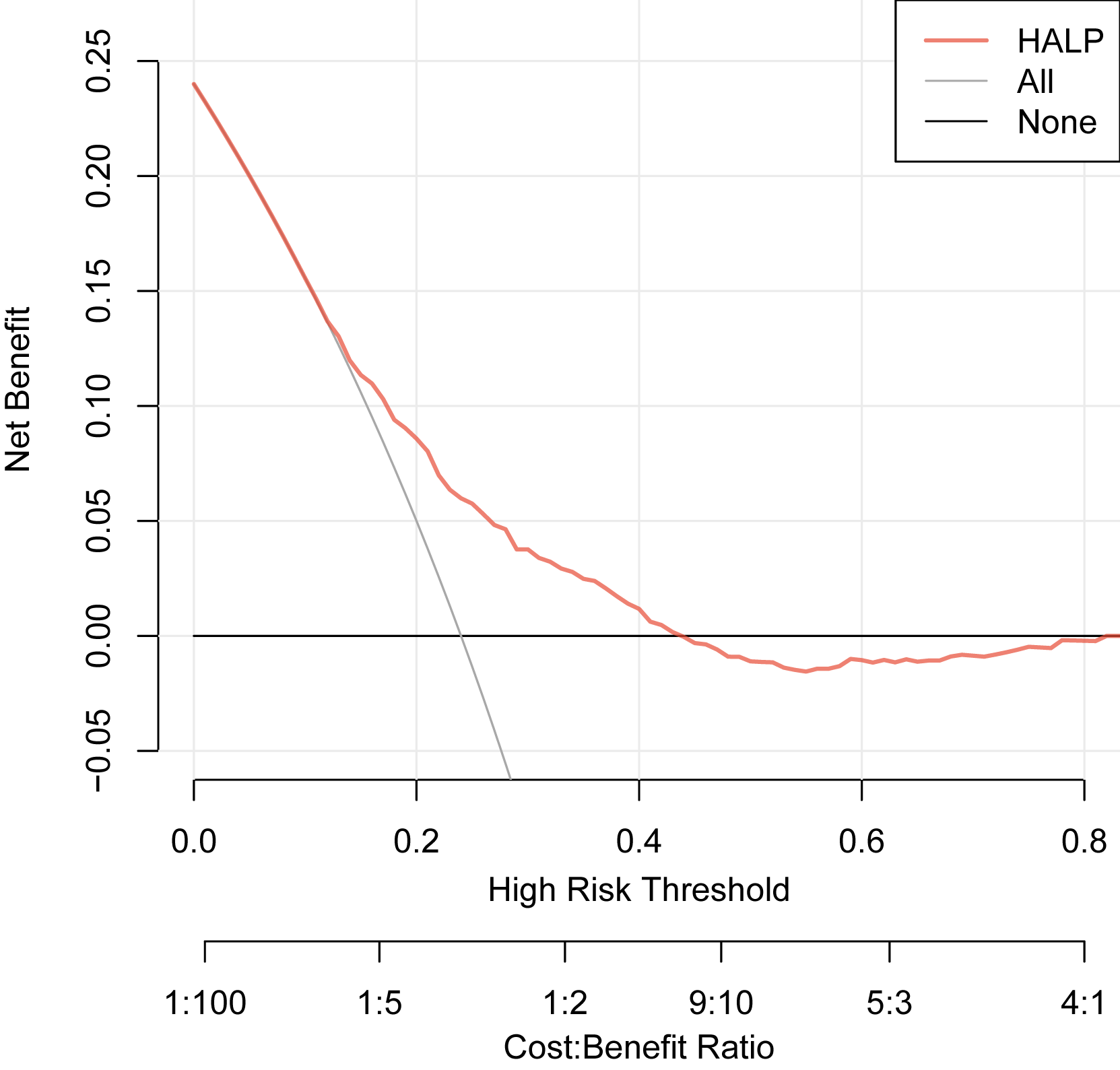

Supplement: Supplementary Figure 1 — Decision curve analysis of HALP score prediction model to estimate DR. [file Image_1.tif]

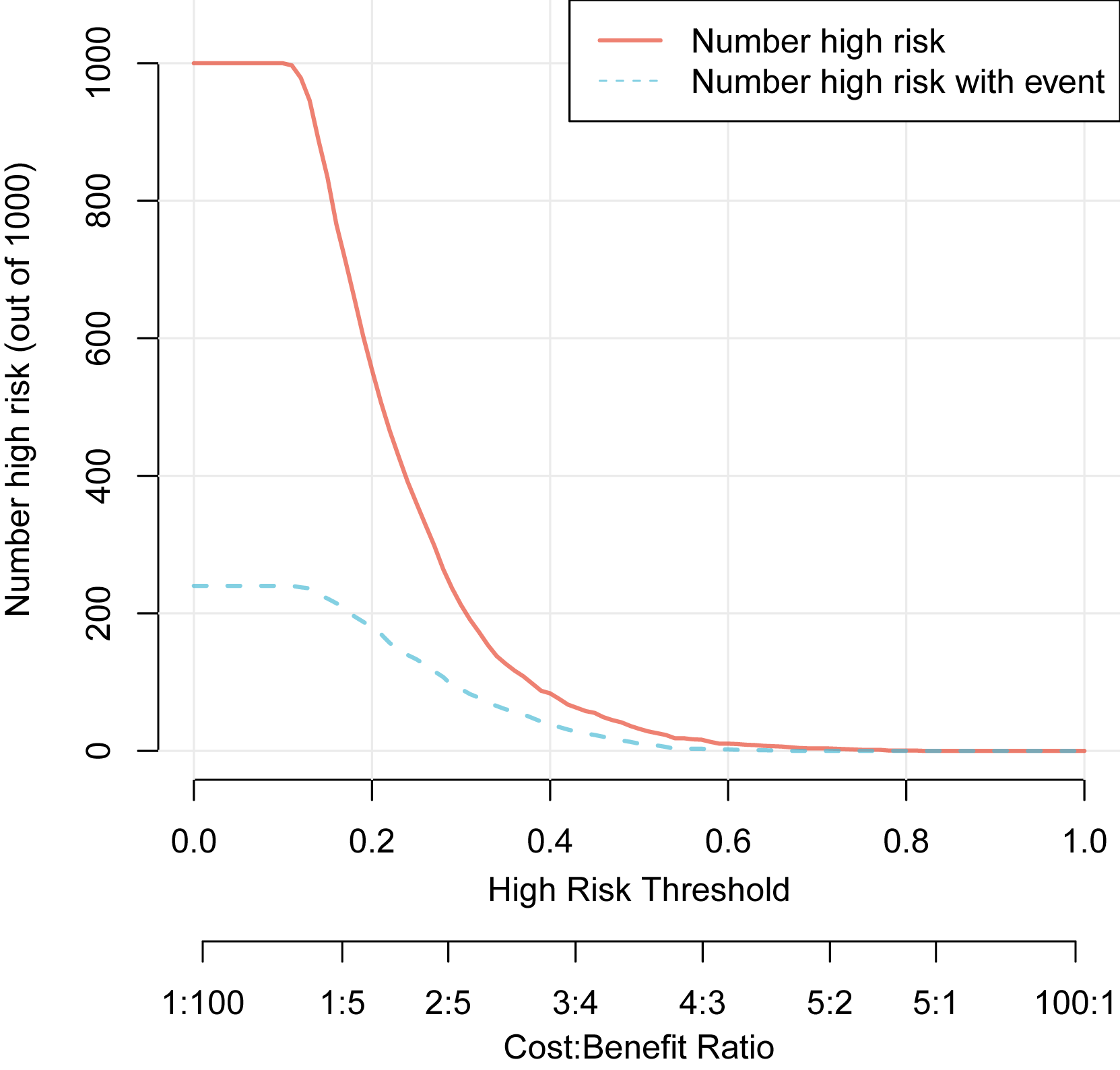

Supplement: Supplementary Figure 2 — Clinical impact curve of HALP score. [file Image_2.tif]
